# Presenting symptoms in inflammatory bowel disease: descriptive analysis of a community-based inception cohort

**DOI:** 10.1186/s12876-019-0963-7

**Published:** 2019-04-02

**Authors:** Bryce Perler, Ryan Ungaro, Grayson Baird, Meaghan Mallette, Renee Bright, Samir Shah, Jason Shapiro, Bruce E. Sands

**Affiliations:** 1 Department of Medicine, Warren Alpert Medical School of Brown University, Providence, USA; 2 Dr. Henry D. Janowitz Division of Gastroenterology, Icahn School of Medicine at Mount Sinai, New York, USA; 3 Lifespan Biostatistics Core, Rhode Island Hospital, Providence, USA; 4 Department of Gastroenterology, Warren Alpert Medical School of Brown University, Providence, USA; 5 Department of Pediatric Gastroenterology, Warren Alpert Medical School of Brown University, Providence, USA

**Keywords:** Inflammatory bowel disease, Crohn’s disease, Ulcerative colitis, Principal component analysis, Ocean State Crohn’s and Colitis Area Registry, Presenting symptoms

## Abstract

**Background:**

Few data are currently available on the initial presenting symptoms of patients with inflammatory bowel disease (IBD).

**Methods:**

We evaluated the initial symptom presentation of patients with IBD in the Ocean State Crohn’s and Colitis Area Registry (OSCCAR), a community-based inception cohort that enrolled Rhode Island IBD patients at time of diagnosis with longitudinal follow up. A 41-question symptom inventory was administered at time of enrollment to capture symptoms experienced during the 4 weeks preceding diagnosis of IBD. Frequencies of presenting symptoms were calculated. Principal component analysis (PCA) with promax rotation was used to examine possible symptom profiles among Crohn’s disease (CD) and ulcerative colitis (UC) patients, respectively. Using the Scree plot, the 4-component solution was found to be optimal for both CD and UC.

**Results:**

A total of 233 CD and 150 UC patients were included. The most common presenting symptoms in CD were tiredness/fatigue (80.6%) and abdominal pain (80.4%) while passage of blood with bowel movements (BM) (86.6%) and loose/watery BMs (86.5%) were most common in UC. The 5 symptoms with greatest differences between UC and CD were passage of blood with BM (UC 86.6%/CD 45.3%), urgent BM (UC 82.5%/CD 63.9%), passage of mucus with BM (UC 67.7%/CD 36.9%), passage of blood from the anus (UC 59.7%/CD 32.1%), and anxiety about distance from bathroom (UC 59%/CD 38.7%). The PCA analysis yielded a 4 symptom components solution for CD and UC.

**Conclusion:**

The most common presenting symptoms in CD are fatigue and abdominal pain while in UC bloody BM and diarrhea are most common. Distinct symptom phenotypes are seen with PCA analysis. Our study demonstrates symptomatic similarities and differences between CD and UC and suggests that patients may also be classified by symptom phenotype at time of diagnosis.

**Electronic supplementary material:**

The online version of this article (10.1186/s12876-019-0963-7) contains supplementary material, which is available to authorized users.

## Background

Inflammatory bowel disease (IBD) is a group of inflammatory gastrointestinal (GI) disorders that are categorized into two major types – ulcerative colitis (UC) and Crohn disease (CD) [1]. There are few published data on the initial presenting symptoms of patients with IBD. Little is known about whether specific symptoms present together or if specific symptom phenotypes correlate with underlying disease classification.

Prominent symptoms in CD often include abdominal pain, diarrhea, weight loss and fatigue [2, 3]. One study looked at symptoms throughout the disease course of IBD and found that the two most common symptoms were diarrhea and fatigue [4]. However, this study did not explore symptoms at initial disease presentation. Another study described clinical characteristics, incidence, natural history, and symptomatic presentation prior to diagnosis in a pediatric population but focused on extra-intestinal manifestations (EIMs) of IBD at the time of diagnosis. The two most common EIMs at presentation were joint pain (20% in CD and 14% in UC) and oral ulcerations (13% in CD and 6% in UC) [5]. Other studies have explored symptoms associated with IBD after the diagnosis had already been established and typically later in the disease course [6–11].

We sought to examine symptom frequency and patterns at time of diagnosis of IBD. In addition, we sought to explore whether certain symptoms occurred more commonly together, and whether such associated symptom clusters were found in either CD or UC.

## Methods

### Patient population

The Ocean State Crohn’s and Colitis Area Registry (OSCCAR) is a community-based prospective IBD inception cohort established in Rhode Island with recruitment occurring between 2008 and 2013. A total of 408 patients were enrolled in the registry. The registry was established in Rhode Island because it is both a small and diverse state. The goal of the OSCCAR cohort was to increase understanding of IBD epidemiology, clinical presentation, disease course, and outcomes [12].

Newly diagnosed adult and pediatric IBD patients who resided in Rhode Island were referred for OSCCAR enrollment by their gastroenterologist or colorectal surgeon [12]. Diagnosis of UC, CD and indeterminate colitis were confirmed using symptom, endoscopic, radiologic and histologic criteria established by the National Institutes of Diabetes and Digestive and Kidney Diseases (NIDDK) IBD Genetic Consortium (Additional file 1) [13]. At the time of initial intake, an extensive interview and chart review were performed to collect demographic and clinical data. A prospective 41-question comprehensive symptom inventory was administered at enrollment to capture symptoms experienced during the 4 weeks preceding diagnosis of IBD (Additional file 2). This symptom inventory was previously developed by combining items from IBD severity indices including the Mayo Index [14], the UCDAI [15], the Seo Index [16], the Ulcerative Colitis Clinical score [17], the Simple Clinical Colitis Activity Index [18] and the St. Mark’s Index [19] along with symptoms mentioned frequently in IBD patient focus groups [20]. Disease location was defined using the Montreal classification [21]. Patients were excluded if they had indeterminate colitis, insufficient data to confirm IBD diagnosis, or did not complete the symptom inventory.

### Statistical analysis

Frequencies of presenting symptoms were calculated for CD and UC patients. Additionally, symptom frequencies were calculated based on CD disease location and UC disease extent. Principal component analysis (PCA) with promax rotation was used to examine possible symptom components among CD and UC patients. All analyses were conducted using SAS Software 9.4 (SAS Inc., Cary, NC) using FREQ, GLIMMIX, and FACTOR procedures.

## Results

A total of 233 CD and 150 UC patients were included in this study. Of the 408 initially enrolled patients, 12 patients did not have sufficient clinical, histologic, or laboratory data to confirm the diagnosis of IBD and 2 patients had indeterminate colitis. Additionally, 11 patients did not fill out the symptom inventory at the time of initial intake.

**Fig. 1:**
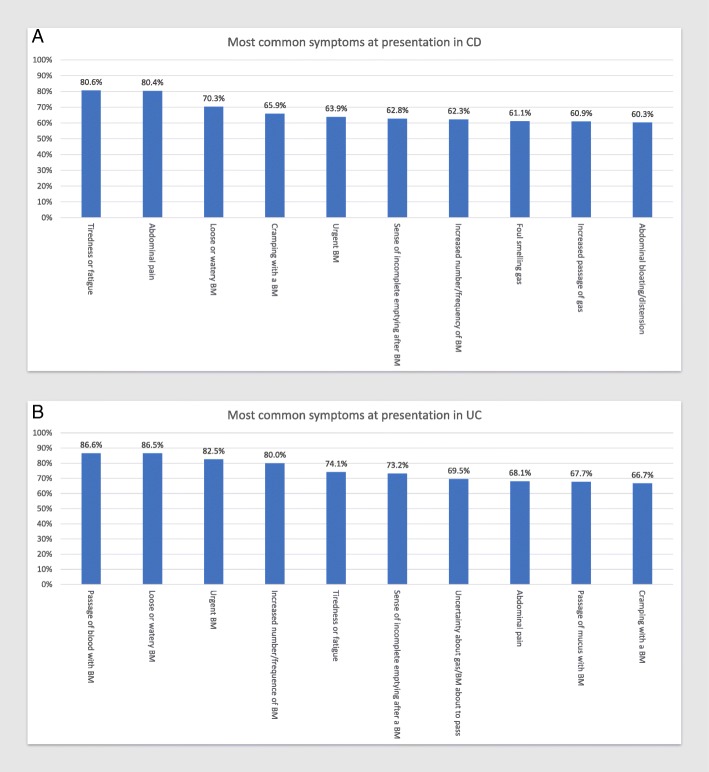
The 10 most common presenting symptoms in CD (**a**) and UC (**b**) captured using the 41-question symptom inventory

The two most common presenting symptoms in CD were tiredness/fatigue and abdominal pain while passage of blood with bowel movements (BM) and loose/watery BMs were most common in UC (Fig. 1a and b). The 5 symptoms with greatest differences between UC and CD were passage of blood with BM (UC 86.6%/CD 45.3%), urgent BM (UC 82.5%/CD 63.9%), passage of mucus with BM (UC 67.7%/CD 36.9%), passage of blood from the anus (UC 59.7%/CD 32.1%), and anxiety about distance from bathroom (UC 59%/CD 38.7%).

**Fig. 2:**
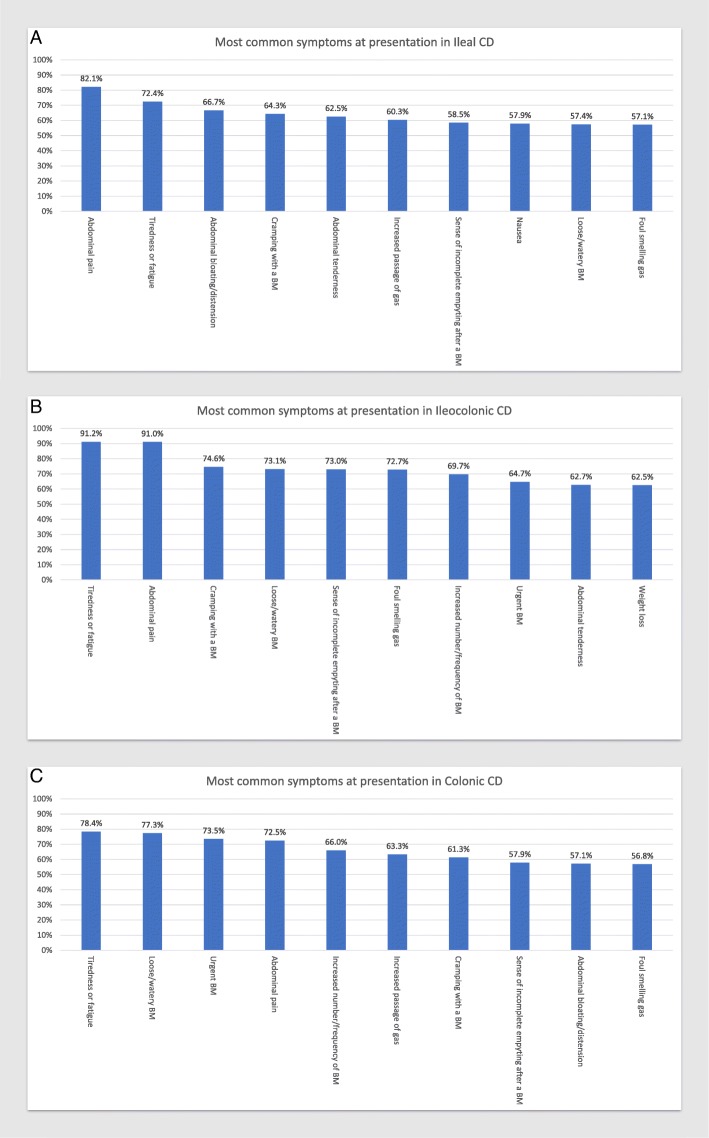
The 10 most common presenting symptoms in Ileal CD (**a**), Ileocolonic CD (**b**), and Colonic CD (**c**) captured using the 41-question symptom inventory

**Fig. 3:**
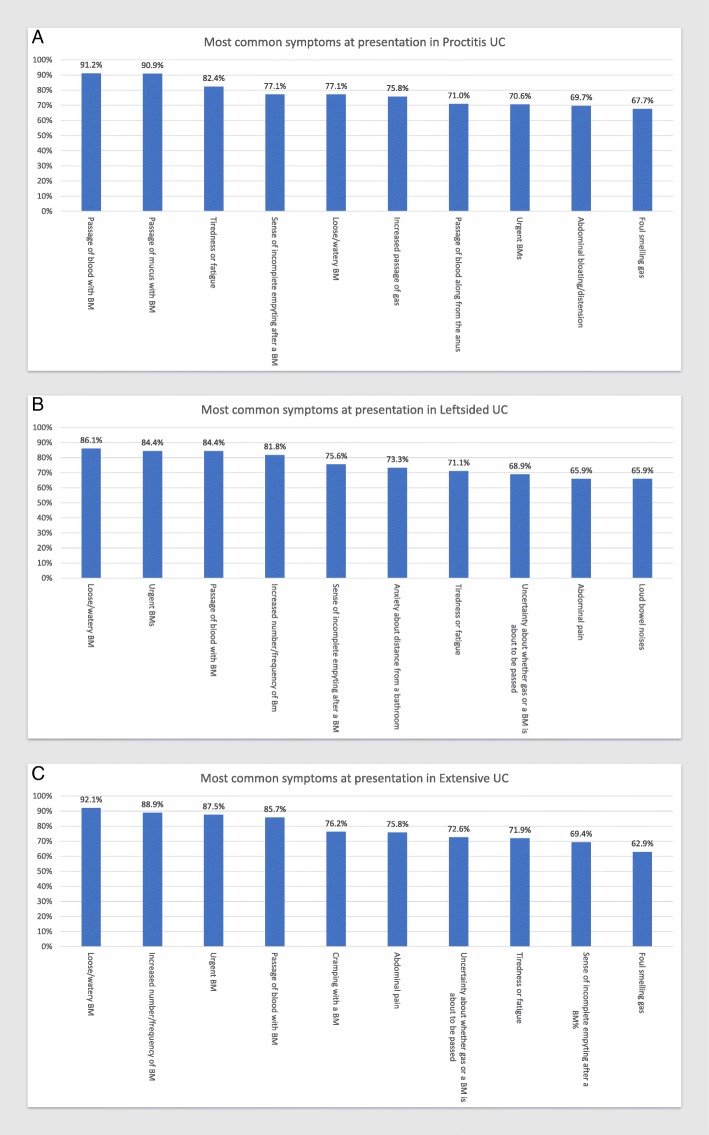
The 10 most common presenting symptoms in Proctitis UC (**a**), Leftsided UC (**b**), and Extensive UC (**c**) captured using the 41-question symptom inventory

When examining disease location by Montreal classification, colonic CD had somewhat different presenting symptoms compared to ileal and ileocolonic CD. Symptoms related to bowel movement consistency were more common in colonic disease. The most common presenting symptoms in CD based on disease location at time of diagnoses were abdominal pain (82.14%) and tiredness/fatigue (72.41%) for ileal CD (Fig. 2a), tiredness/fatigue (91.18%) and abdominal pain (91.04%) for ileocolonic CD (Fig. 2b). and tiredness/ fatigue (78.35%) and loose or watery BMs (77.32%) for colonic CD (Fig. 2c).The most common presenting symptoms in UC based on disease extent at time of diagnosis are passage of blood with BM (91.18%) and passage of mucus with BM (90.91%) for proctitis (Fig. 3a), loose or watery BMs (86.05%) and urgent BMs (84.44%) for left-sided disease (Fig. 3b), and loose or water BMs (92.06%) and increased number or frequency of BMs (88.89%) for extensive/pancolitis (Fig. 3c).

**Fig. 4:**
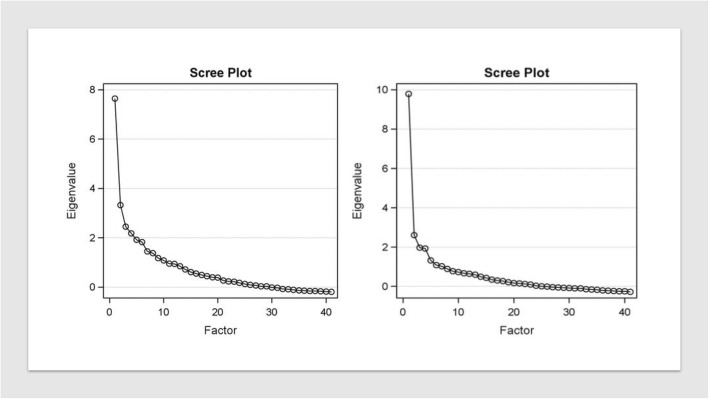
Scree plots of UC (left) and CD (right). Eigen values denote variance accounted for by a linear combination. As can been seen in the left panel (UC) and right panel (CD), the amount of variance accounted is greatest for the first component, then the second, followed by the third and fourth, but diminishes greatly thereafter; this reveals the 4-component solution is the best

The PCA using the Scree plot found a 4-component solution to be optimal for both CD and UC using a loading threshold of 0.30 (Fig. 4). The symptom profiles for CD included components with predominantly 1) bowel frequency and abdominal discomfort symptoms, 2) systemic/extraintestinal symptoms, 3) anorectal symptoms, and 4) upper abdominal symptoms (Table 1). The 4 components for UC were 1) bowel frequency and abdominal discomfort symptoms, 2) systemic/extraintestinal symptoms, 3) anorectal symptoms and 4) incontinence and flatus symptoms (Table 1). When UC and CD data were combined, no interpretable component solutions were found. This was also true when performing a cluster analysis, where UC and CD were not found to cluster separately nor were consistent and interpretable symptom clusters found. This suggests that symptomatology between the two diseases significantly overlaps.

**Table 1:** Symptom components for CD and UC

CD		UC	
Bowel frequency and abdominal discomfort symptom component	urgent BM, increased number/frequency of BMs, loud bowel noises, anxiety about distance from bathroom, cramping with BM, increased passage of gas, uncertainty about whether gas or BM is about to be passed, foul smelling gas, loose/watery BM, sense of incomplete emptying after BM, BM at night, abdominal tenderness, abdominal bloating/distension.	Bowel frequency and abdominal discomfort symptom component	weight loss, loose/watery BM, increased number/frequency of BM, urgent BM, decreased appetite, cramping with BM, anxiety about distance to bathroom, loud bowel noises, feeling full before a full meal has been eaten, abdominal pain, BM at night, abdominal tenderness, and fever
Systemic/ extraintesinal symptom component	painful joints, swollen joints, achiness, sore/painful eyes, lightheadedness, red eyes, insomnia/trouble sleeping, and nausea	Systemic/ extraintesinal symptom component	painful joints, achiness, lower back pain, swollen joints, and insomnia/trouble sleeping
Anorectal symptom component	passage of blood along anus, incontinence/leakage of stool, drainage/seepage from around anal area, pain around the anal area between BM, passage of mucus with BM, pain around anal area with BM	Anorectal symptom component	pain around the anal area with BM, pain around anal area in between BM, hard stools/difficult to pass, abdominal bloating/distention, sore/painful eyes*,* cold sweats/chills, and passage of blood along the anus
Upper abdominal symptom component	decreased appetite, feeling full before a full meal has been eaten, weight loss, vomiting, fever, and cold sweats/chills	Incontinence and flatus symptom component	incontinence/leakage of stool, foul smelling gas, drainage/seepage from around anal area, and increased passaged of gas

Table displays the 4 symptom components generate by PCA for both CD and UC with the symptoms that loaded within each component. Of the 4 components, 3 are the same for CD and UC, while the last 1 for each is distinct

## Discussion

In a cohort of newly diagnosed IBD patients, we found the two most common presenting symptoms in CD were fatigue and abdominal pain while in UC bloody BM and diarrhea were most common. Previous studies have demonstrated common presenting symptoms of abdominal pain, diarrhea and fatigue but none have captured as extensive a symptom inventory as our study [2–4]. Additionally, our population was exclusively a community-based inception cohort in the modern era while the majority of the previous studies were older. The most common symptoms in CD and UC that we observed that were not previously captured in inception cohorts include loose or watery BMs, cramping with a BM, urgent BMs, sense of incomplete emptying after a BM, increased number or frequency of BMs, foul smelling gas, increased passage of gas, abdominal distention or bloating, uncertainty about gas or BM about to pass, and passage of mucus with BM. In addition, we were able to explore initial symptoms based on CD location and extent of UC. These findings provide a more detailed picture of the predominant symptoms often encountered at diagnosis in IBD.

We observed distinct symptom profiles using PCA analysis. The components seen in PCA were very similar for both CD and UC. Three of the 4 domains are common between the two conditions, including bowel frequency and abdominal discomfort symptoms, systemic/extra-intestinal symptoms and anorectal symptoms. The only domains that were different were upper abdominal symptoms for CD and incontinence and flatus symptoms for UC. This underscores the significant overlap of symptoms between CD and UC.

Limitations of our study include utilization of pre-set symptom descriptions as opposed to both a dimensional quantitative assessment (spectrum of intensity instead of binary yes/no. Because about half of all patients did not answer one or more symptoms questions, PCA results only reflect those who completed the questionnaire completely (*n* = 183) and thus these PCA results may not be generalizable. This could be why an interpretable and consistent solution was not found using cluster analyses. Each model generated different results with no specific cluster being associated to any disease class. In addition, the PCA results reveal some questions do not load to components while others load with more than one component. These results should be interpreted with caution as they are provided as description only. Larger samples are needed to provide more robust PCA results. Finally, caution should be exercised when comparing the rates of symptoms between CD and UC. Although our study shows which symptoms are experienced by CD and UC patients, given the differences in base rate of each disease observed in our sample the positive predictive value of individual symptoms or clusters is not meaningful for this study. This highlights the limitation of using isolated symptoms to distinguish between diagnoses.

Future directions would include collecting symptom inventory on more patients to determine if a specific symptom cluster was associated with disease phenotype, quality of life, or outcomes over time. This could provide a framework for an additional method of classifying patients with IBD based on symptom phenotype. Our study demonstrates symptomatic similarities and differences between CD and UC and suggests that patients may also be classified by distinct symptom phenotype components.

## Conclusion

This study is the most extensive presenting symptom inventory of IBD patients at time of diagnosis to date. The most common presenting symptoms in CD are fatigue and abdominal pain while in UC bloody BM and diarrhea are most common. PCA generated 4 distinct symptom phenotypes with 3 of the 4 domains being common between CD and UC, including bowel frequency and abdominal discomfort symptoms, systemic/extra-intestinal symptoms and anorectal symptoms. The fourth domain for the 2 conditions were different which included upper abdominal symptoms for CD and incontinence and flatus symptoms for UC. Our study demonstrates symptomatic similarities and differences between CD and UC and suggests that patients may also be classified by symptom phenotype at time of diagnosis. Additionally, with the substantial heterogeneity in presentation of IBD, diagnosis and treatment can be delayed. This study provides further data on presenting IBD symptoms and symptom phenotypes which may assist in future diagnostic and phenotyping studies.

## Additional files

Additional file 1: NIDDK IBD Genetic Consortium Phenotype Operating Manual. The Phenotype Operating Manual helped to establish a standardized protocol to help identify and diagnose IBD then further categorize into UC, CD or Indeterminate Colitis based on symptoms, endoscopic, radiologic and histologic evidence. (DOCX 1474 kb)

Additional file 2: Symptom Inventory. The symptom inventory combines BID severity indices with symptoms mentioned frequently in IBD patient focus groups. It is a comprehensive questionnaire that encompasses both luminal and extraluminal symptoms. (DOCX 873 kb)

## Abbreviations

BM: Bowel movement
CD: Crohn’s disease
IBD: Inflammatory bowel disease
OSCCAR: Ocean State Crohn’s and Colitis Area Registry
PCA: Principal component analysis
UC: Ulcerative colitis
