# Correction to: Presenting symptoms in inflammatory bowel disease: descriptive analysis of a community-based inception cohort

**DOI:** 10.1186/s12876-020-01526-2

**Published:** 2020-12-03

**Authors:** Bryce K. Perler, Ryan Ungaro, Grayson Baird, Meaghan Mallette, Renee Bright, Samir Shah, Jason Shapiro, Bruce E. Sands

**Affiliations:** 1 Department of Medicine, Warren Alpert Medical School of Brown University, Providence, USA; 2 Dr. Henry D. Janowitz Division of Gastroenterology, Icahn School of Medicine At Mount Sinai, New York, USA; 3 Lifespan Biostatistics Core, Rhode Island Hospital, Providence, USA; 4 Department of Gastroenterology, Warren Alpert Medical School of Brown University, Providence, USA; 5 Department of Pediatric Gastroenterology, Warren Alpert Medical School of Brown University, Providence, USA

## Correction to: BMCGastroenterology (2019) 19:47 10.1186/s12876-019-0963-7

Following publication of the original article [1], the authors identified an error in one of the authors’ names. The correct author name should be Bryce K. Perler.
