# Supplementary figures and images for: Presenting symptoms in inflammatory bowel disease: descriptive analysis of a community-based inception cohort
Source: BMC Gastroenterol. 2019 Apr 2;19:47. doi: 10.1186/s12876-019-0963-7 (PMC6446285; doi:10.1186/s12876-019-0963-7)

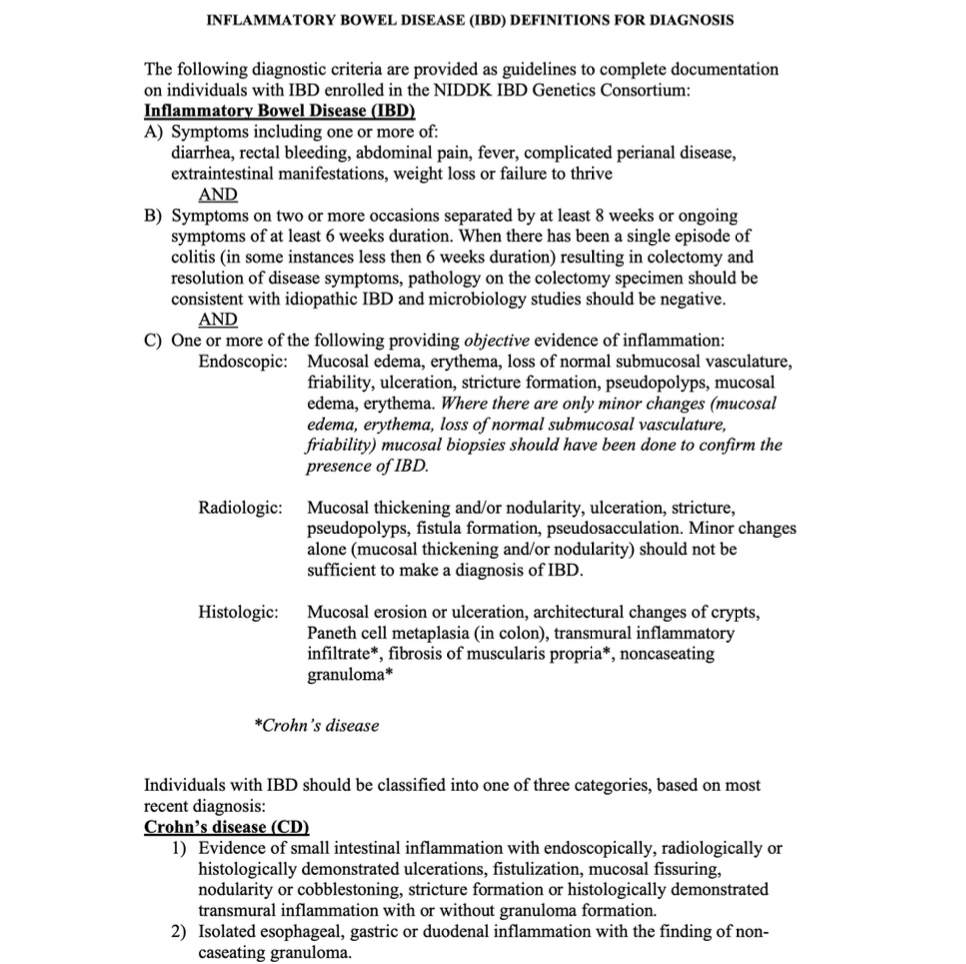


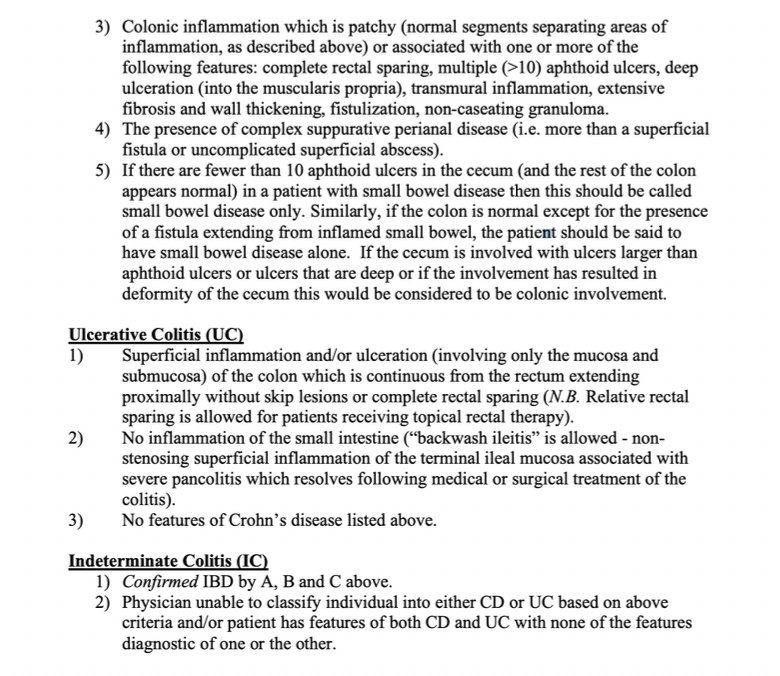

Supplement: Supplementary file 1 — NIDDK IBD Genetic Consortium Phenotype Operating Manual. The Phenotype Operating Manual helped to establish a standardized protocol to help identify and diagnose IBD then further categorize into UC, CD or Indeterminate Colitis based on symptoms, endoscopic, radiologic and histologic evidence. (DOCX 1474 kb) [file 12876_2019_963_MOESM1_ESM.docx]

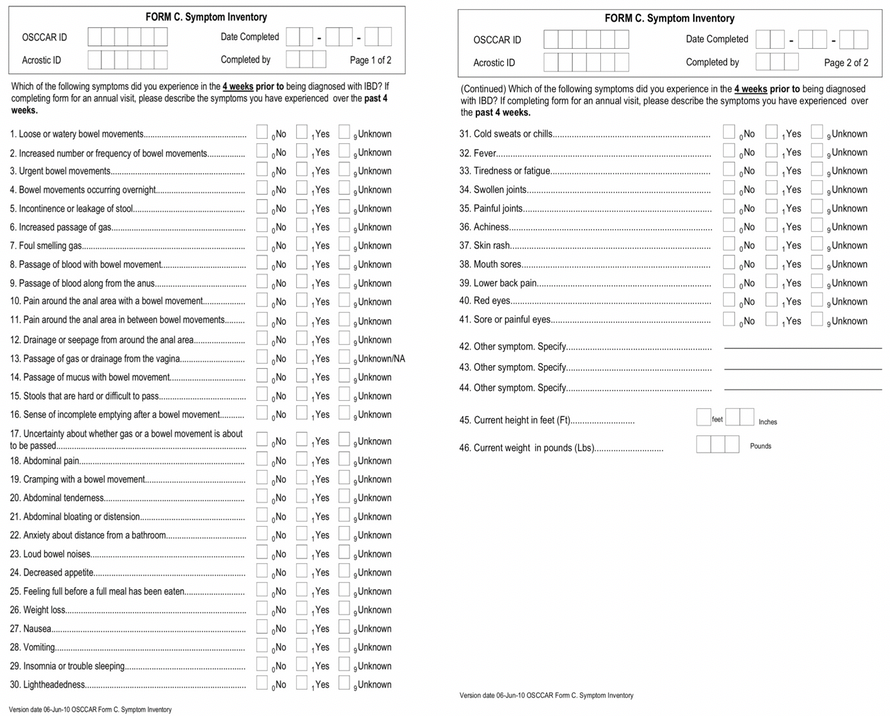

Supplement: Supplementary file 2 — Symptom Inventory. The symptom inventory combines BID severity indices with symptoms mentioned frequently in IBD patient focus groups. It is a comprehensive questionnaire that encompasses both luminal and extraluminal symptoms. (DOCX 873 kb) [file 12876_2019_963_MOESM2_ESM.docx]
